# At the Feet of the Fortress: Analysis of Inka Period (ca. AD 1430-1536) Archaeofaunal Assemblages from Residential Unit 1 (RU1), Pucara de Tilcara (Jujuy, Argentina)

**DOI:** 10.1371/journal.pone.0163766

**Published:** 2016-10-12

**Authors:** Carlos Raúl Belotti López de Medina, Lautaro López Geronazzo, Clarisa Otero

**Affiliations:** 1 Instituto de las Culturas (IDECU), Universidad de Buenos Aires, CONICET, Museo Etnográfico J. B. Ambrosetti (FFyL), Ciudad Autónoma de Buenos Aires, Argentina; 2 Instituto de Ecorregiones Andinas (INECOA), Universidad Nacional de Jujuy, CONICET, San Salvador de Jujuy, Jujuy Province, Argentina; 3 Instituto de Ecorregiones Andinas (INECOA), Universidad Nacional de Jujuy, CONICET, San Salvador de Jujuy, Jujuy Province, Argentina; University of Hawaii at Manoa, UNITED STATES

## Abstract

This paper reports the results of a zooarchaeological analysis conducted on the occupation layer of a compound structure (Residential Unit 1) of the Pucara de Tilcara archaeological site (Jujuy Province, northwestern Argentina). Its occupation span extends between the 13^th^ and 15^th^ centuries AD, but evidence diagnostic of the Inka Period (AD 1430–1536) is predominant. Residential Unit 1 was a house-workshop that hosted specialized crafts like metallurgy and lapidary during the Inka Period. It was proposed in previous works that artisans living at Pucara de Tilcara were provisioned with agropastoral products by the Inka administration. This paper aims to test that hypothesis against the zooarchaeological evidence of Residential Unit 1. Three variables were used as proxies for state-sponsored distribution: taxonomic diversity (family and species ranks), and skeletal and age profiles of the predominant zoological family (Camelidae) in the assemblage. The results show a high degree of continuity with the regional record, characterized by a herding-hunting strategy focused on domestic and wild species of Camelidae and a mixed mortality pattern. The skeletal profile shows a strong and negative correlation with the desiccation potential of elements, which could be indicative of local production of *chalona*. Overall, faunal evidence does not show any sign of centralized distribution.

## Introduction

The onset of the second millennium AD marks a widespread development of middle range polities across the Circumpuna sub-area (South-Central Andes), including the Quebrada de Humahuaca in northwestern Argentina. These organizations have been compared to chiefdoms and federations [1], heterarchies [2], communal organizations [3] and ranked corporate kin-groups [4,5]. Supra-domestic division of labor increased in tandem with political complexity (*e*.*g*. the scale and complexity of bronze metallurgy increased at many semiarid valleys in northwestern Argentina beginning ca. AD 1000) [1,6]. Division of labor also involved the control of diverse ecological zones (verticality) by groups ranging from lineages to ethnic groups [1,7]. The Inka Empire, or *Tawantinsuyu*, reinforced these trends after the conquest of the South-Central Andes at the beginning of the 15^th^ century.

Change in social complexity brings up the issue of how agropastoral products were allocated within the population. Distribution of food and drink during reciprocal services, communal works and labor tributes was a traditional practice of Andean societies [8,9]. The Inka amplified this system and even intensified agricultural production to sustain corvée laborers, drafted soldiers and retainers [10,11]. However, distribution did not exclude self-sufficiency as even attached specialists received shares of land to grow their own food; actually, full-time attached specialists were a late development [11].

Zooarchaeology provides a set of analytical tools and models for research on distribution, since specialization and hierarchy often affect the acquisition of animal goods [12–14]. This paper presents a case study on the procurement of faunal resources by craft specialists during the Inka Period, based on the analysis of faunal remains from a late pre-Hispanic house-workshop: Residential Unit 1 of the *Pucara de Tilcara* archaeological site, in the *Quebrada de Humahuaca* region of Jujuy Province. To date, two mechanisms for the procurement of faunal resources by central settlements in this region during the second millennium AD have been hypothesized: 1) provisioning by specialist transhumant herders belonging to the same kinship-networks or domestic groups as the consumers beginning ca. AD 1200 [7], and; 2) elite or state-sponsored redistribution among craft specialists during the Inka Period (AD 1430–1536) [15]. The aim of this paper is to test the latter model.

## Background

**Fig 1:**
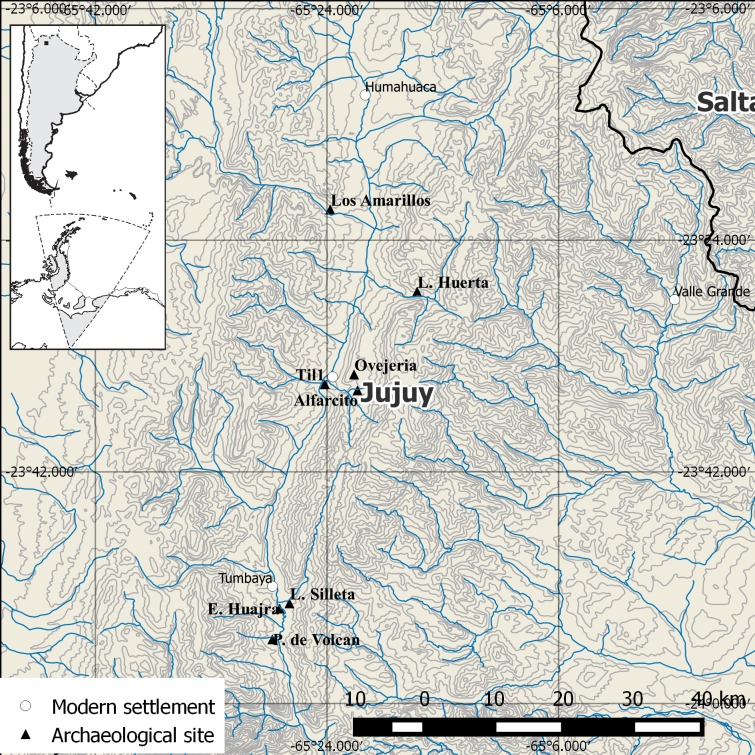
Map of Humahuaca and location of the archaeological sites and localities mentioned in the text. The present map was created with QuantumGIS based on data from the Instituto Geográfico Nacional de la República Argentina (http://www.ign.gob.ar/) and Natural Earth (http://www.naturalearthdata.com/).

The Quebrada de Humahuaca is a narrow and semiarid intermontane valley (Fig 1), whose maximally 120 km long by 3 km wide main corridor is formed by the Grande River. Its northern limit extends to the confluence of the *Tres Cruces* and *Cóndor* streams at Azul Pampa, approximately 3600 m asl. Its southern limit extends to the modern town of *Volcán* at approximately 2000 m asl [16]. The Quebrada de Humahuaca is flanked by two ecological zones: 1) the southern segment of the Andean plateau (*Puna*) to the west; and, 2) the Sub-Andean hills and eastern forests (*yungas*) to the east.

The pre-Columbian occupation of northwestern Argentina follows an evolutionary course common to the South Central-Andes (Table 1): initial settlement by hunter-gatherers minimally by 10,600–8400 BP, judging by the earliest dates from sites like Inca Cueva 4 and Huachichocana III [17]; subsistence intensification leading to domestication (Archaic Period, ca. 9000–1000 BC) [18,19]; the establishment of sedentary agropastoral lifeways (Formative Period, ca. 1000 BC–AD 900 in Humahuaca) [20,21], with political organizations characterized as tribal or non-ranked [16,20]; and, followed by the Regional Developments I period (ca. AD 900–1200), characterized by the earliest evidence of social ranking [16,22].

**Table 1:** Cultural sequence of the agropastoralist stage of Humahuaca, based on the synthesis proposed by Nielsen [4,16].

Archaeological Period	Major trends
Inka (ca. AD 1430–1536)	• Construction of large agricultural and storage facilities by the state. • Demographic relocations, including previously uninhabited sectors.
Regional Developments II (ca. AD 1200–1430)	• Political integration of various settlements. • Ranked corporate kin-groups • Warfare and demographic concentration in the main valley continue. • Economic intensification. • Caravan trade • Large settlements on defensible terrains.
Regional Developments I (ca. AD 900–1200)	• Factionalist competition and individual ranking. • Warfare and demographic concentration in the main valley. • Caravan trade • Economic intensification
Formative (ca. 1000 BC–AD 900)	• Diffusion of agropastoral sedentary lifeways. • Household level organization. • Population loosely distributed across the main valley and tributaries.

Subsequent archaeological periods include Regional Developments II (ca. AD 1200–1430) and Inka (ca. AD 1430–1536) [5,16]. The former is characterized by a florescence of complex pre-state polities, usually modeled after chiefdoms or ranked corporate kin-groups [5,23]. These polities were eventually subordinated by *Tawantinsuyu* after *ca*. AD 1430 [16]. *Tawantinsuyu* was an expansive state whose political economy was based primarily on imposed corvée labor from peasant communities [9–11,24].

### Pucara de Tilcara, Residential Unit 1

**Fig 2:**
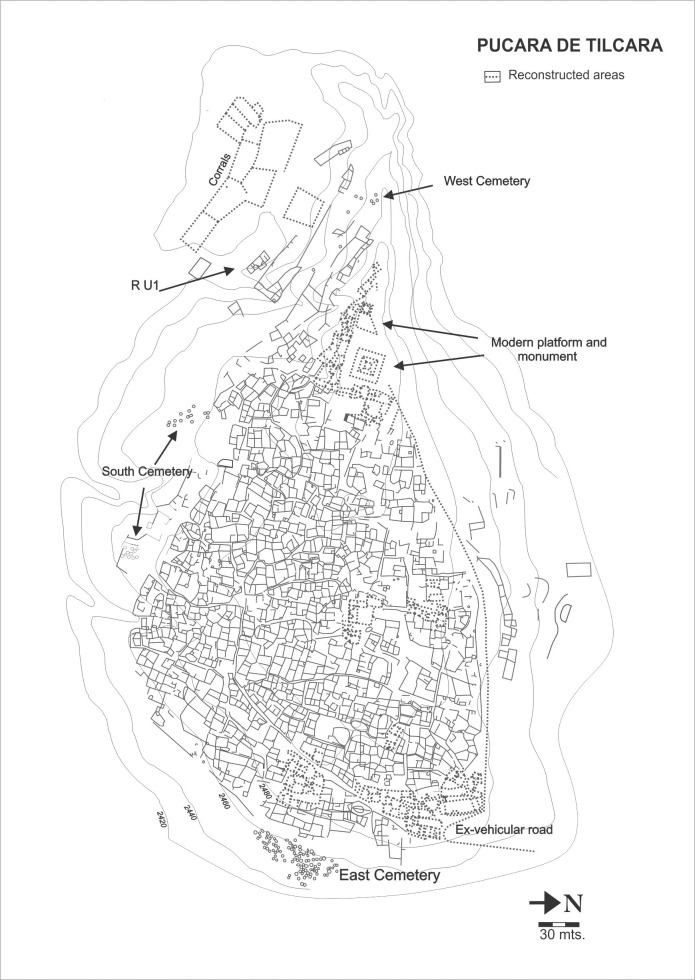
Layout of the Pucara de Tilcara settlement and location of Residential Unit 1. Source: Otero, C. 2015. Distribución y consumo de cerámica Inca en el Pucará de Tilcara (Quebrada de Humahuaca, Argentina). Chungara 47(3), p. 402 (Figura 1), http://dx.doi.org/10.4067/S0717-73562015005000019.

Pucara de Tilcara (Til1) is an iconic site in northwestern Argentina and was the subject of several research projects throughout the last century [15,25–37]. It is located next to the confluence of the *Huasamayo* and *Grande* rivers, south of the modern town of Tilcara in the central segment of the main valley of Humahuaca (Fig 1) (23°35'11.10"S, 65°24'10.40"W). The site covers approximately 17.5 ha, extends over an 80 m high promontory (Fig 2) [37], and could have been occupied by up to 1500 people. Settlement began on the lower hillslopes of the promontory, eventually spreading upwards to the peak around the late 13^th^ century AD [38,39].

Til1 is believed to have been the center of a middle-range polity [1] and a regional hub for distribution of diverse goods [15] during the Regional Developments II period. The site became a center for specialized crafts and may have been the paramount administrative center of the Humahuaca *wamani* (province) under the Inka Empire. Evidence of metallurgy, as well as of manufacture of non-utilitarian goods like pendants, plaques and ritual figurines (*illas*) made of different kinds of valves and stones (onyx, limestone, flint and alabaster), and, to a lesser extent, pottery and weaving were recovered from more than 50 groups of structures [40]. Occupation of Til1 ended during the 16^th^ century, some decades after the fall of *Tawantinsuyu*.

Our zooarchaeological analysis is part of an ongoing research project directed by Clarisa Otero at the *Instituto Interdisciplinario de Tilcara* (*Universidad de Buenos Aires*), which continues on previous studies undertaken by Myriam Tarragó. In addition to other lines of research, Otero has reconstructed archaeological contexts of artifacts from the Pucara which are curated in museum collections [40]. It has been demonstrated that many households at the site were devoted to multi-craft production under the Inka [15]. The Imperial administration would have been in charge of providing artisans with food and raw materials [15] judging by the large agricultural facilities constructed at Humahuaca and nearby areas during the Inka Period [1,16]. Part of the food consumed at Til1 could have been produced at the agricultural complex of the Alfarcito-Ovejería area (Fig 1), and on the valley floor of the Grande River [15].

**Fig 3:**
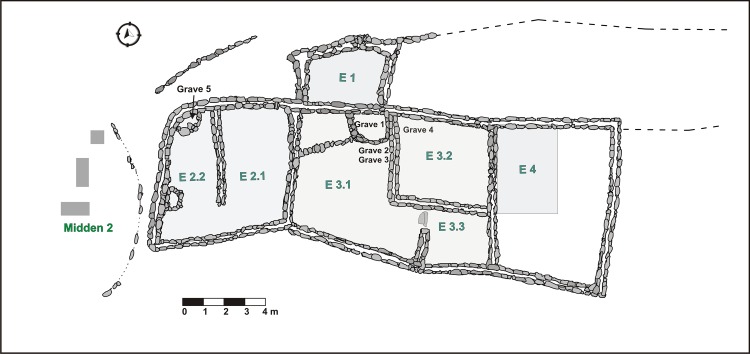
Residential Unit 1 (RU1), Midden 2 and excavated areas (gray).

Our research focuses on the faunal collection from RU1, a compound structure located on the southwestern slopes of the promontory (Fig 2 and Fig 3). Tarragó conducted earlier fieldwork on this section of Til1 between 1988 and 1992, including the excavation of a large area of RU1 and an associated trash-dump (Midden 2) [35,36]. The southwestern slopes were selected because they were outside the restored and touristic sector of Til1, and neither were disturbed by previous research. Since 2006, Otero has been continuing fieldwork on RU1 [15,41].

RU1 consists of a series of conjoining enclosures built around a central courtyard (Enclosure 3.1, Fig 3) with a surface area of approximately 160 m^2^. Most of RU1 was excavated down to the sterile horizon. Occupation strata (floors) were identified in all enclosures. Living floors were dated to *ca*. AD 1200–1500 [38] based on recent radiocarbon dates (Table 2), which include both Regional Developments II and Inka period occupations. However, analysis of radiocarbon dates and artefactual evidence indicate the predominance of Inka phase occupation [16], probably as a result of continuous settlement and floor sweeping, creating a true palimpsest (*sensu* Bailey [42]). The floors contained evidence of *in situ* activities such as food preparation, knapping and use of lithic tools, pottery and metallurgy [15,28,34,36,41,43–45]. RU1 was characterized as a multi-functional space–a workshop-house–attached to the Inka administrators [15].

**Table 2:** Radiocarbon dates from Til1-RU1.

Date	C14 dates (BP)	Calibrated dates (AD) 1σ	Calibrated dates (AD) 2σ
LP 2240	450±40	• 1441–1499 (61.1%) • 1599–1610 (7.1%)	• 1425–1513 (72.5%) • 1547–1623 (22.9%)
LP 2231	450±50	• 1436–1504 (54.7%) • 1591–1615 (13.5%)	• 1419–1520 (65.3%) • 1537–1626 (30.1%)
LP 2191	450±60	• 1431–1508 (51.3%) • 1585–1619 (16.9%)	• 1418–1627 (95.4%)
LP 2467	470±50	• 1425–1498 (65.9%) • 1602–1607 (2.3%)	• 1405–1513 (76.2%) • 1545–1624 (19.2%)
AA88342	510±46	• 1414–1455 (68.2%)	• 1395–1499 (94.3%) • 1599–1609 (1.1%)
AA88340	512±41	• 1418–1452 (68.2%)	• 1397–1484 (95.4%)
AA88341	561±42	• 1400–1438 (68.2%)	• 1323–1346 (7.6%) • 1388–1452 (87.8%)
AA89445	635±52	• 1311–1359 (43.2%) • 1380–1409 (25.0%)	• 1293–1425 (95.4%)
LP 247	800±50	• 1225–1286 (68.2%)	• 1182–1313 (90.6%) • 1358–1381 (4.8%)
LP 536	910±70	• 1048–1083 (15.1%) • 1139–1230 (49.0%) • 1250–1261 (4.0%)	• 1033–1273 (95.4%)

LP: Laboratorio de tritio y radiocarbono (LATYR, UNLP, CONICET). AA: NSF Arizona AMS Facility.

RU1 is especially apt for exploratory research on the modes of procurement of faunal resources by craft specialists during the latest pre-Hispanic periods. Previous zooarchaeological research on Til1 amounts to a preliminary report on a sample from Midden 2 [44]. Only 127 bone fragments from a small sample of 343 specimens were identified, and these were predominantly camelids (NISP = 122, 96%), with five specimens of small rodents. The Camelidae sub-assemblage showed a marked predominance of appendicular skeletal elements from mature animals (88% based on epiphyseal fusion). Midden 2 is believed to have been accumulated in part by the inhabitants of Residential Unit 1.

### Animal exploitation at Humahuaca

Agropastoral societies from the South-Central Andes were highly specialized on three camelid species [46–50]: the wild southern vicuña (*Vicugna vicugna vicugna*) [51] and northern guanaco (*Lama guanicoe cacsilensis*) [52], and the domesticated llama (*Lama glama*) [53]. The current distribution of vicuña is restricted to elevations above 3500 m asl (Andean Plateau). The alpaca (*Vicugna pacos*) [53] is ill adapted to arid environments and thus is believed to have been absent from the study area [48]. Camelids were the main source of animal protein, grease and raw materials [49]; secondary exploitation included wool from wild and domestic animals, and llamas served as draft animals for caravan trade [7,46,54]. Camelid specimens contribute from 50 to 100% of identified bone specimens at Humahuaca agropastoral sites [55–73].

The mixed exploitation of wild and domestic species was characterized as a herding-hunting strategy by Yacobaccio *et al*. [47] and its ubiquity has been explained as a risk-aversion and livestock conservation strategy [47,74,75]. Pre-Hispanic pastoralism was monospecific [46,48] and domestic herds are vulnerable to yearly fluctuations due to environmental stress [76,77]. This pattern was reinforced by other practices, including intentional hybridization [78] and shearing of wild camelids [46].

Social organization of herding and hunting varied across different ecological zones and through time. At least two modes of indirect procurement of faunal resources–or camelids, to be precise- have been proposed for the central settlements of the main Humahuaca valley. First, Nielsen posited the existence of specialist transhumant herders since the Regional Developments II Period [7]. They would have settled at the higher tracts of tributary ravines next to the Puna, and in the Puna itself, in order to graze animals during the wet season (summer). They could have hunted wild camelids for immediate or delayed consumption (dried meat) during their stays. Pastoralists would travel down to the main valley during the harvest season, where herds could have been fed crop stubble and riverine vegetation. Nielsen hypothesized that herders belonged to the same nested kinship groups (*ayllus*) and domestic groups as the dwellers of the central settlements, and that exchange among them would have been enforced by reciprocal obligations [7].

Second, Otero proposed that the craft specialists of Til1 could have been provided with food by the Inka state. In addition, Mengoni Goñalons proposed that camelid carcasses and cuts were distributed from different origins based on three Inka assemblages in Humahuaca and the Calchaquí valley [66]. State-sponsored distribution of food among tributaries and retainers, at least for the duration of their service, was a well-established institution of *Tawantinsuyu* [10,24]. The Empire also allocated raw materials like wool among tributary households for production of finished goods [10,11]. Evidence of this system in northwestern Argentina includes large agricultural and storage facilities dated to the Inka Period [15,16,79]. Historical accounts affirm that the state and clergy possessed large herds of llamas (*capac llama*), which were in the care of tributary workers and specialist retainers [10,80,81]. In addition, the Inka exerted some regulation on hunting [46,82]. Our working hypothesis is that the dwellers of RU1 were provided with food by the imperial administration.

## Materials and Methods

### Residential Unit 1

Fieldwork at RU1 combined test-pits and area excavations, covering approximately 127 m^2^ or 79% of the total compound area (Fig 3). All sediment was sieved through screens with a mesh size of 1/8-inch (3.2 mm). Three main stratigraphic units were identified at each room: 1) *post-occupation fillings*, which included remains of collapsed walls that had rolled downhill from structures located above RU1; 2) *living floors*; and, 3) *underlying sterile layers*. Smaller features such as hearths, postholes, and trash middens were also found; most were part of the floor layers with others from later occupation.

A total of 1804 bone specimens were recovered from every stratigraphic unit of RU1 excavated between 1988 and 2009. This analysis is restricted to specimens from the occupation layer (floor) (NSP = 1171). The floor assemblage was probably the outcome of casual discard following preparation and consumption. Most specimens are less than 100 mm in length (%NSP = 95); larger and more obtrusive fragments may have been discarded outside living areas. The horizontal distribution of bones is irregular, with highest concentrations occurring at the R2 group of enclosures (NSP/m^2^ = 22.5), followed in decreasing order by N4 (6.2), R3 (3.4), and R1 (0.1). Excavation records for Room 2.2 report bone concentrations near the structure walls. This pattern reinforces previous inferences [15] on the functional differentiation of rooms (see also [83]).

Table 3 tallies the number of analyzed specimens by body-size class (NSP) and Table 4 tallies the number of identified specimens (NISP) by taxon and size class. Size classes follow the scale published by Izeta for Northwestern Argentina [75,84]. This scale includes five classes: 1. small, *e*.*g*. *Ctenomys* sp. <0.5 kg; 2. small, *e*.*g*. *Lagidium viscacia* ~1.6 kg, *Chaetophractus vellerosus* ~0.8 kg; 3. medium, *e*.*g*. *Pterocnemia pennata* ~30 kg; 4. big, *e*.*g*. *V*. *vicugna vicugna* ~50 kg, *Lama* sp. ~70–120 kg; 9. indeterminate size.

**Table 3:** Number of analyzed specimens (NSP) from the living floors by body-size class.

Body size	NSP	
	Non-identified	Identified
1–2	2	-
2	-	2
2–3	1	-
3–4	309	16
4	92	413
9	336	-
Total	740	431

**Table 4:** Number of taxonomically identifiable specimens (NISP) from the living floors.

Taxon	Body size	NISP
Artiodactyla:		
Artiodactyla indet.	3–4	16
Artiodactyla indet.	4	57
Camelidae	4	354
Cervidae	4	2
Rodentia:		
Chinchillidae (*Lagidium viscacia*)	2	2
	Total	431

Table 5 details the number of identified specimens (NISP) of the Camelidae sub-assemblage. The sub-assemblage is composed of 354 specimens. The highest Minimum Number of Individuals (MNI) was six (left humerus). Three camelid morphotypes were morphometrically identified: *Lama* sp. NISP = 20, *L*. *glama* NISP = 30 and *V*. *vicugna vicugna* NISP = 14. The maximum MNI values for specific morphotypes were: cf. *L*. *glama* MNI = 3 (left scaphoid), cf. *V*. *vicugna vicugna* MNI = 1 (right calcaneus). The *Lama* sp. morphotype includes specimens whose measures cluster with *L*. *guanicoe cacsilensis* and the smallest reference measurements for *L*. *glama*, and which therefore could belong to any of the two species.

**Table 5:** Number of taxonomically identifiable specimens (NISP) of the Camelidae sub-assemblage by morphotype.

Taxon	NISP
Camelidae indet.	290
*Lama* sp.	20
*Lama glama*	30
*Vicugna vicugna vicugna*	14
Total	354

### Methods

Distribution and exchange are important topics in the zooarchaeology of complex societies [12,14]. Three aspects of the zooarchaeological record are useful for approaching these topics across a wide range of archaeological settings [12,14]: taxonomic diversity, age profiles, and frequency of skeletal elements. However, none of these variables is straightforward [85]; they require an approach using models and frames of reference relevant to the area and period under study. Fortunately, there is a growing body of data on Andean pastoralism from which to draw ideal patterns that can be compared with zooarchaeological data.

Usually, zooarchaeological assemblages in northwestern Argentina are characterized by low taxonomic diversity (predominance of Camelidae or of indeterminate artiodactyls) (see also [48,49]). However, demographic increase and population concentration in large settlements, craft specialization and Inkan subjugation could all possibly affect these measurements. For example, small prey items accumulated through foraging in rural settings (*e*.*g*. armadillos, rodents) could be missing from nucleated villages like Til1. On the other hand, the Inka purportedly introduced exploitation of two domestic species to northwestern Argentina, the cuy or guinea pig (*Cavia porcellus*) and the muscovy duck (*Cairina moschata*) [86]. Also, purported Inkan regulations on the hunting of wild camelids [10] could have resulted in a decreasing frequency of guanacos and vicuñas between the Regional Developments II and the Inka periods.

In terms of age-profiles, Wapnish and Hesse [87] posit that in a consuming economy where animals are acquired from pastoralists, mortality profiles should exhibit a relative abundance of market-age animals (after weight gain tapers off), or a marked seasonality if animals are provided by transhumant herders. Conversely, production sites should exhibit higher rates of very young and senile animals [87]. A similar approach could be applicable to Andean assemblages. Yacobaccio published a series of epiphyseal fusion profiles from modern Puna pastoralists [88] who practice a mixed exploitation of herds (production of fiber and meat). These profiles show survival rates over 80% for the first three years of development and could serve as a model for production sites of transhumant pastoralists.

Regarding consumption at central settlements of complex polities, faunal samples from Chavín de Huántar in the Central Andes (Janabarriu phase ca. 400–200 BC) show different mortality patterns [89]. High-status Sector D is characterized by a predominance of camelids under 36 months old, while 60% of animals were sacrificed after four years of age at the low-status Sector A. Mengoni Goñalons [66] observed the preferential killing of immature camelids (2–3 years old) at Humahuaca sites of the Regional Developments II and Inka periods. On the other hand, Til1 exhibits abundant evidence for caravan trade [15], which would suggest a secondary production profile (predominance of old animals) [90,91]. As for wild animals, historical sources state that the Inka restricted vicuña hunting to the capture of living animals for shearing (*chacu*), and only the culling of old animals was allowed [46].

In terms of skeletal profiles, a skewed distribution of animal parts may be a potential proxy for exchange/distribution as long as consumers are provided with butchered anatomical units instead of complete carcasses (*e*.*g*. the selection of meatier or optimal anatomical portions, as well as spatial discontinuity for butchering stages) [14]. Preferential consumption of dried meat is another potential signature of distribution [89]. Drying is a traditional practice throughout the Andes, allowing for storage and delayed consumption of meat. The earliest model for distribution of dried meat was the *ch’arki* effect posited by Miller [89,92], which distinguishes high-altitude production locations from lower-altitude consumer sites. The former should exhibit a higher ratio of heads and lower limbs (metapodials, phalanges), while the latter should exhibit higher frequencies of post-cranial axial bones and of meatier upper limbs. This model has been criticized on the basis that there are various kinds of dried meat, which are termed differently throughout the Andes. For instance, Stahl distinguishes two main kinds [93]: 1) *chalona*, equivalent to Miller *ch’arki*, which is not deboned during preparation; 2) *ch’arki sensu stricto*, which is deboned. Skeletal or body parts profiles produced by *chalona* consumption should exhibit a positive correlation with utility and a negative correlation with structural bone density, while *ch’arki* consumption would be archaeologically invisible. Another useful frame of reference is the Drying Index published by De Nigris and Mengoni Goñalons [94], which ranks skeletal elements according to their potential for drying.

Lab analyses were carried out at the Casanova Archaeological Museum, following methods and techniques detailed previously [95]. Production of primary data included (S1 File): anatomical and taxonomic identification of bone specimens based on morphological criteria [96–101] (only specimens identified to order level or lower taxonomic rank were counted as identified); fusion of secondary ossification centers of endochondral bones [102–104] (age determination based on teeth was precluded for the lack of preserved tooth-rows); taphonomic signatures [105–107]; and, morphometric measurements of a sample of Camelidae postcranial elements [104,108] (S2 File). Measurements were compared to modern specimens from osteological collections [68,84,109,110] through a set of multivariate morphometric analyses following protocols by Menegaz *et al*. [111] and Izeta [84,112]. As a result, sampled archaeological specimens were classified into modern specific morphotypes.

Quantification of primary data included the following measures: 1) **Number of Specimens** (NSP, analyzed specimens) and %NSP; 2) **Number of Identifiable Specimens** (NISP, order or lower taxonomic level) and %NISP. Additional measures for the Camelidae sub-assemblage included: 1) Minimum Number of Elements (MNE), based on counts of long-bone ends and shaft fractions [113], and counts of shaft landmarks [106,114]; 2) Minimum Number of Individuals (MNI) [115,116]; 3) Minimum Animal Units (MAU, %MAU) for whole elements, derived from MNE [106,117,118]; 4) and a second set of %MAU values derived from Camelidae bone-density scan-sites [93].

Taxonomic diversity in the family rank is described using the NTaxa and equitability indices [119,120]. In addition, individual based rarefaction curves (based on zoological family) were produced for RU1 and other Regional Developments II—Inka residential assemblages with PAST (Paleontological Statistics, http://folk.uio.no/ohammer/past/) [121–123].

Additional analyses were undertaken for camelid specimens. First, epiphyseal data were classified into three fusion groups [66,88,124] based on published sequences [102,104] and analyzed with zooaRch [125]. Second, a series of Spearman’s *rho* correlation analyses was conducted on the skeletal profile of Camelidae using Statistica 7.0 (Statsoft). These include correlations between scan-site %MAU vs. shape-adjusted volume density (VD_SA_) [93,126]; and, element %MAU vs. Food Utility Index (FUI) [127], Medullary Cavity Volume (MCV) [128], and Drying Index (DI) [94].

## Results

### Taxonomic Diversity

Ungulates are predominant (Camelidae NISP = 354, Artiodactyla indet. NISP = 73) and the majority of unidentified fragments belongs to size-classes 3–4 (NSP = 401). Small vertebrates are almost absent from the assemblage (NSP = 5), which could be partially attributed to screen mesh-size. According to Thomas [119,129], there is an estimated loss of up to 64% for vertebrates weighing >700 g between 1/8-inch and 1/16-inch meshes. However, factoring NSP for size-classes 1–2 by the estimated loss gives an additional NSP of only nine specimens.

**Fig 4:**
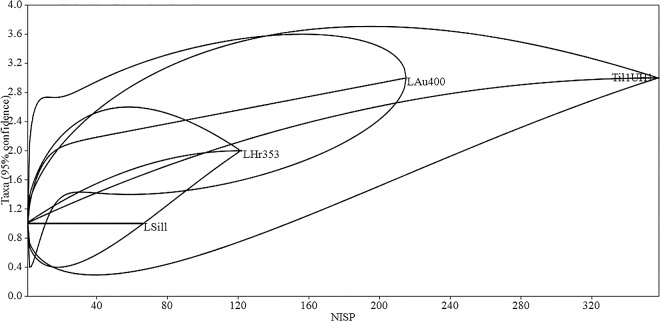
Rarefaction curves for Residential Unit 1, Los Amarillos unit 400, La Huerta enclosure 353 and La Silleta.

Camelidae is the dominant taxon at the family rank (%NISP 99.5), which is reflected also by richness (NTaxa 3) and the Equitability Index (V’ 0.06). A predominance of Camelidae is common in almost every agropastoralist assemblage from northwestern Argentina (*ex supra*). It is similar to Midden 2 in this respect, which also exhibited a marked predominance of Camelidae. Fig 4 plots rarefaction curves (family) for RU1 and other faunal assemblages recovered from residential contexts spanning the Regional Developments II–Inka periods: Los Amarillos unit 400 (LAu400) [59], La Huerta enclosure 353 (LHr353) [67], and La Silleta (LSill) [73].

The RU1 rarefaction suggest a low equitability for RU1 in comparison to LAu400 and LH353. In addition, interpolation with LAu400 is indicative of lower richness. However, the 95% Confidence Intervals of the RU1 rarefaction overlap with those of the remaining assemblages. Finally, none of the species that could be diagnostic of state-sponsored distribution during the Inka regime were found at RU1 (*e*.*g*. *C*. *porcellus*, *C*. *moschata*). In sum, the RU1 floor assemblage is taxonomically similar to other agropastoral sites from Humahuaca since the Regional Developments II period.

Co-occurrence of both llama and vicuña is indicative of the aforementioned herding-hunting strategy. Vicuña bones indicate also the exploitation of the Humahuaca higher ravines and the Puna. Other evidence of hunting includes obsidian projectile points and waste flakes found on the living floor of RU1 and sourced to Puna outcrops [43].

### Camelidae

The analysis of mortality and skeletal profiles focuses on Camelidae, as it is the most abundant taxon. However, morphometric identification of specific morphotypes was restricted to a small sample of the Camelidae sub-assemblage due to taphonomic processes affecting the preservation of measurable elements (NISP = 64, %NISP = 18). Therefore, quantification was necessarily restricted to the family taxonomic rank in order to obtain an assemblage amenable to statistical analyses. This is the standard procedure of zooarchaeological studies carried out in the South-Central Andes, but a methodological corollary is that most quantifications will average the outcome of hunting and pastoralism as the Camelidae assemblage would include bones from domestic (*L*. *glama*) and wild (*V*. *vicugna*) species.

Camelid bones were heavily fragmented (%NISP broken bones = 65); the only unbroken specimens were phalanges, carpals and tarsals. High levels of fragmentation are a common occurrence in faunal assemblages from northwestern Argentina and, although non-cultural agents cannot be dismissed, it is plausible that at least some breakage was due to butchering. Yacobaccio *et al*. [90] observed that Highland pastoralists boiled most carcasses, breaking long bones into three or four pieces to fit into pots (pot-sizing). This practice facilitates the addition of bone marrow to available food resources [90]. The same practice was recorded by Miller, who observed how upper limb bones could be broken in as much as five or six pieces [92]. Another hypothesis for breakage is fat extraction by boiling bone splinters [130]. Optimal extraction could be reached by boiling ≤5 cm long splinters for approximately two hours [131].

Fragmentation ratios for green and non-green breaks from limb bones (NISP:MNE) [105,132] range between 2.5 (Tibia) to 3.7 (Femur). For long bones, there were more fractures on green bones (NISP = 57) than non-green or columnar breaks (NISP = 36). The average length of splinters from green bones is 67.35 mm (Std. dev. 27.83 mm). It is relevant for both the pot-sizing and fat extraction hypotheses that 5170 potsherds were recovered at RU1. Partially refited vessels (n = 250) were classified as cooking and serving wares with mouth-diameters between 35–40 and 60 cm [15]. There is insufficient evidence to attribute fragmentation to either hypothesis, given that bone splinters are small, yet above the optimal size for fat-extraction. Nevertheless, intensive exploitation of in-bone nutrients seems plausible.

The assemblage exhibits post-mortem modifications that in many cases are diagnostic of human behavior. Butchering marks were recorded on 23 specimens (%NISP 6.5). This figure is low in comparison to experimental studies (*e*.*g*. [133]) and many archaeological cases (*e*.*g*. [106,134]), but falls within the range exhibited by other domestic assemblages from agropastoral sites of northwestern Argentina (*e*.*g*. [55,75,95]). It has been observed that butchering mark frequencies are highly variable and that one relevant factor could be intensive fragmentation (*ex supra*) [135]. Cutmarks were compared to actualistic studies for interpretation [118,136,137]. Six specimens present filleting marks (scapulae NISP 5, humerus NISP 1), three specimens show disarticulation marks (radius-ulna NISP 1, tibia 1). Indeterminate cutmarks were recorded for ten specimens, including ribs, radius-ulna and basipodia. Flake-scars and percussion pits were recorded for five long-bone splinters (radius-ulna, metatarsals and humerus). Filleting marks could be indicative of *in situ* defleshing for preparation and consumption.

#### Age-profile

**Fig 5:**
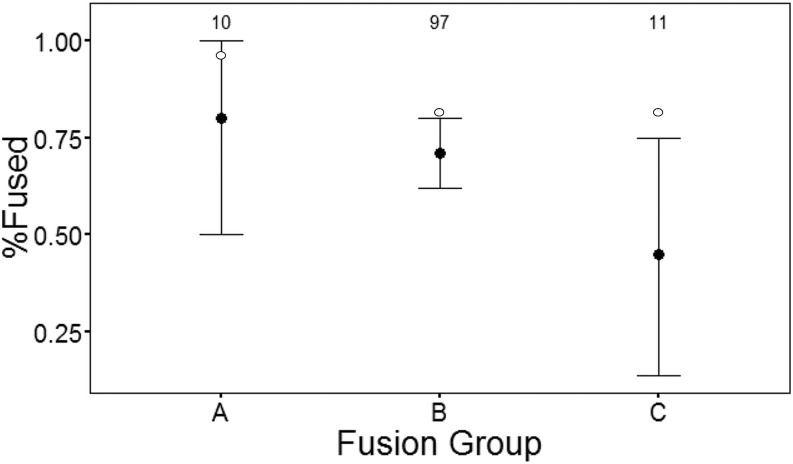
Survival curve for the RU1 Camelidae sub-assemblage. Black dots: frequency of fused bones by fusion group (survival). Whiskers: confidence intervals for survival. White dots: survival values from modern pastoral sites.

Table 6 tallies the number of specimens by stage of epiphyseal fusion and Fig 5 plots the resulting survivorship curve, including modern values for the fiber-meat mixed strategy [88]. The profile shows a high rate of camelid survival at least until the second year (71%), followed by a marked decrease toward the third year, which is an opposite trend to natural mortality in modern herds [77] and below that of the mixed fiber-meat strategy [88].

**Table 6:** Number of identified specimens (NISP) by epiphyseal fusion and fusion group, and survivorship (%NISP).

Fusion group	Elements	NISP		%NISP Fused (Survivorship)
		Unfused	Fused	
A (< 12–18 months old)	Innominate (acetabulum), distal humerus, distal scapulae.	2	8	80
B (< 18–36 months old)	Proximal phalanges, calcaneus (tuberosity), distal tibia, distal metapodial.	28	69	71
C (< 36–48 months old)	Distal radius-ulna, proximal humerus, proximal femur, distal femur, proximal tibia.	6	5	45

A similar but more accentuated decline of survivorship was observed at Esquina de Huajra, which Mengoni Goñalons interpreted as a young or immature-oriented mortality profile resulting from a surplus of young animals [66]. Selection of domestic animals for reproduction or for training as draft animals takes places between the animal’s second and third years. A survival rate of 45% for group C could reflect an equilibrium between secondary and primary production, *i*.*e*. a mixed strategy, but more biased towards meat production. However, the confidence interval for group C of RU1 is too wide and the resulting survival rate could be a sampling artifact (Type I error).

The bulk ratio of fused elements (%NISP 69) is below that of Midden 2 (%NISP 88) [44], pointing to a moderately lower degree of livestock conservation. However, it must be observed that the Midden 2 report does not classify specimens by fusion groups and comparison is thus unwarranted.

#### Skeletal profile

**Fig 6:**
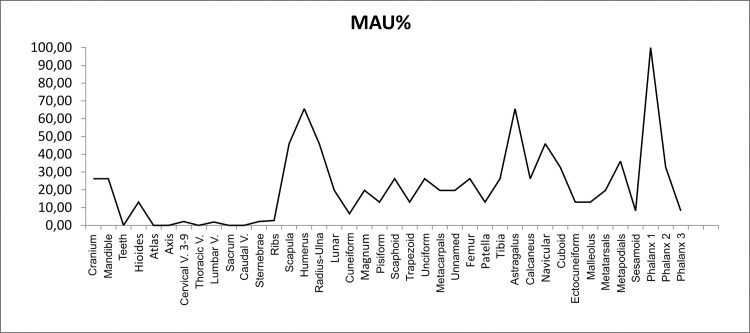
Camelidae Skeletal profile (%MAU).

Table 7 tallies the abundance of skeletal elements (NISP, MNE, MNI, and MAU) and Fig 6 plots the standardized Minimum Animal Units (%MAU) for Camelidae. A higher frequency of limb bones is clear, especially for the first phalanx, humerus, radio-ulna, metapodial, and tarsus. This pattern is the opposite of that observed at modern pastoralist sites [90,138], but it is not uncommon for archaeofaunas from northwestern Argentina [95]. In addition, it does not conform to the *ch’arki* effect suggested by Miller [89,92]. Overabundance of phalanges at RU1 could be attributed to transport of these elements as riders of meatier limb bones [130].

**Table 7:** Skeletal profile of the Camelidae sub-assemblage.

Element	NISP	MNE	MNI	MAU	%MAU
Cranium	12	2	2	2.0	26.2
Mandible	4	2	1	2.0	26.2
Teeth	3	3	1	0.0	0.0
Hyoid	1	1	1	1.0	13.1
Atlas	–	0	0	0.0	0.0
Axis	–	0	0	0.0	0.0
Cervical V.	2	1	1	0.2	2.2
Thoracic V.	–	0	0	0.0	0.0
Lumbar V.	3	1	1	0.1	1.9
Sacrum	–	0	0	0.0	0.0
Caudal V.	1	1	1	0.0	0.0
Sternebra	1	1	1	0.2	2.2
Ribs	23	5	1	0.2	2.7
Scapula	14	7	5	3.5	45.9
Innominate	7	3	2	1.5	19.7
Humerus	26	10	6	5.0	65.6
Radius-Ulna	22	7	4	3.5	45.9
Lunar	3	3	2	1.5	19.7
Cuneiform	1	1	1	0.5	6.6
Magnum	4	3	3	1.5	19.7
Pisiform	2	2	1	1.0	13.1
Scaphoid	4	4	3	2.0	26.2
Trapezoid	2	2	1	1.0	13.1
Unciform	4	4	3	2.0	26.2
Metacarpals	4	3	2	1.5	19.7
Femur	15	4	3	2.0	26.2
Patella	2	2	1	1.0	13.1
Tibia	10	4	3	2.0	26.2
Astragalus	12	10	6	5.0	65.6
Calcaneus	6	4	3	2.0	26.2
Navicular	7	7	5	3.5	45.9
Cuboid	5	5	4	2.5	32.8
Ectocuneiform	2	2	1	1.0	13.1
Malleolus	2	2	2	1.0	13.1
Metatarsals	3	3	3	1.5	19.7
Metapodial	46	11	3	2.7	36.1
Sesamoid	10	10	1	0.6	8.2
Phalanx 1	66	61	8	7.6	100.0
Phalanx 2	20	20	4	2.5	32.8
Phalanx 3	5	5	2	0.6	8.2

On a side note, the NISP skeletal profile of RU1 is similar to that of Midden 2; a Spearman’s *r* correlation between them was strong and significant (%NISP by element, *r*_*s*_ = 0.678, p. < 0.01), reinforcing the hypothesis of an interconnected taphonomic history. However, a comparison based on %MAU or %MNE would be preferable to one based on NISP as the latter is influenced by fragmentation, but the original report on Midden 2 includes neither of the former measures.

An intra-bone Spearman r correlation analysis was conducted on scan-site %MAU vs. bone density (VD_SA_) for single elements with > 3 *scan-sites* to monitor for density-mediated attrition [93,126]. The correlations were weak to moderate and non-significant, except for the mandibles (Table 8).

**Table 8:** Spearman correlation (r_s_) of %MAU vs. VDsa (bone density) for elements of the Camelidae sub-assemblage with >3 scan-sites.

Element	*Scan-sites*	r_s_	p. <0,05
Mandible	8	0.866	0.005
Ribs	5	0.224	0.718
Scapula	4	-0.105	0.895
Humerus	5	0.667	0.219
Radius-Ulna	5	-0.087	0.870
Metacarpal	6	-0.507	0.305
Innominate	7	0.493	0.261
Femur	6	0.088	0.870
Tibia	5	-0.154	0.805
Calcaneus	4	-0.775	0.225
Metatarsal	6	-0.414	0.414

Table 9 resumes a series of Spearman’s *r* correlation analyses on %MAU against various utility indices (Food Utility, Marrow Cavity Volume and Drying) and bone density (VDsa). The Food Utility Index (FUI) for non-long bones and the Drying Index (DI) show strong and significant correlations (reverse utility curves). The former is problematic as it includes the highest ranking elements (ribs), which simultaneously are both hard to identify beyond the ordinal level [93] and more sensitive to attritional processes [139]. Marean and Frey propose conducting correlation analyses on long bones counts based on both articular ends and shaft fragments to avoid these issues [139]. The correlation obtained following their protocol was moderate and positive, but non-significant, suggesting an unbiased strategy. Neither the FUI nor the structural density correlations conform to the profiles proposed by Stahl [93] for *chalona* production or consumption sites.

**Table 9:** Spearman *r* (r_s_) correlation analyses of %MAU against shape-adjusted density values and utility indices.

Correlation analysis	*r*_*s*_	p. < 0.05
%MAU vs. FUI (whole skeleton)	-0.38	0.11
%MAU vs. FUI (non long bones)	-0.71	0.02
%MAU vs. FUI (long bones)	0.50	0.31
%MAU vs. MCV	-0.75	0.08
%MAU vs. DI	-0.68	<0.01
scan-site %MAU vs. VDsa	-0.06	0.56

A negative correlation between skeletal profile and Drying Index was observed by Mengoni Goñalons at other assemblages dated to the Inka period [66]. This reverse curve suggests two scenarios: 1. dwellers of RU1 were fed with fresh cuts from animals used to produce both fresh and dried meat, a hypothesis previously suggested by Mengoni Goñalons for other assemblages [66]; and, 2. RU1 produced dried meat to be consumed at another location or by different consumers.

## Discussion

Sociocultural complexity requires the allocation of resources among diverse and specialized social subsystems. This could be accomplished through diverse institutions, from reciprocity and barter to markets and state-sponsored distribution. Market exchange was foreign to the pre-Hispanic Andes and the peasantry usually retained dominion over their means of production under different social formations [9,10]. Even retainers expatriated from their ethnic communities were granted small shares of land for subsistence [11]. Supra-household economic cooperation was attained through different kinds of reciprocity, communal duties, and labor tributes [8,9].

The beneficiary and organizer of reciprocal services or labor tributes was obliged to provide food and drink to Andean laborers [8], and the *Tawantinsuyu* was no exception [10,24]. Production and storage of staple goods to sustain armies, tributaries, and full-time specialists, as well as for ritual and political events, was a top priority for Inka rulers [24]. The state and the church possessed large herds of llamas and alpacas, and the former declared itself the owner of all wild camelids [10,24,81]; domestic camelids were used to produce wool that was later distributed among tributary households for textile production [11], as draft animals for the armies and to produce fresh and dried meat [10]. Vicuñas were captured alive for shearing (*chacu*) from whose wool attached specialists (*aclla*) made fine fabrics (*cumbi*), while guanacos were hunted for production of dried meat [10,46].

State distribution of animal goods or resources existed alongside traditional peasant patterns of property and the exploitation of communal herds (*waccha llama*) [10,81]; day to day subsistence was largely in charge of the peasantry itself. Craft specialists could have been provisioned on a more regular basis by the state, especially if they practiced a complex productive process like metallurgy [11]. It is also possible that specialists continued acquiring faunal resources through traditional kin-networks and reciprocal obligations. As pointed out by deFrance [13], pre-modern complex polities often left traditional subsistence mechanisms barely touched.

Two main modes of distribution of faunal resources have been posited for the larger central settlements of Humahuaca during the Regional Developments II and Inka periods. One suggests reciprocal exchange between specialist transhumant herders and consumers belonging to the same domestic groups or kin-groups, a pattern that should be included into a broader system of vertical control of ecological zones [7]. Another posits the centralized distribution of food-staples and faunal resources among attached specialists during the Inka Period [15].

Zooarchaeology coupled with contextual information and settlement patterns is a potential avenue for understanding the modes of distribution deployed by the Inka state. The faunal assemblage from the living floor of Residential Unit 1 was a potentially informative case-study on the modes of procurement of faunal resources during this period as the architectural compound hosted both domestic and specialized craft activities, the latter under the patronage of the Imperial administration. Three main proxies of distribution and exchange of animal products at urban settings were selected for this work following the relevant literature: taxonomic diversity, skeletal profiles and age-profiles.

Taxonomic diversity at RU1 is low but well within the normal values of agropastoralist sites since the Formative Period across northwestern Argentina. In addition, there is no trace of any domestic species, like *C*. *porcellus*, potentially introduced by the Inka. This suggest a strong techno-economic continuity from previous periods, regardless of the mode of acquisition of faunal resources. The same can be said for other sites subjugated by the Inka, like Pucara de Volcán, Esquina de Huajra and La Huerta [63,64,66]. Nonetheless, sampling problems cannot be discarded as the low equitability of the record implies that marginal taxa could be missed from all but the largest assemblages. Regarding wild camelids, the recovery of obsidian projectile points and waste flakes at RU1 demonstrates exploitation of Puna environments and suggest a strong relationship with transhumant herders. Obsidian knapping together with vicuña faunal remains has been recorded at other sites from intermontane valleys since the beginning of the Formative Period across northwestern Argentina [140,141], pointing again to a strong continuity.

The Camelidae survivorship curve shows rates below those of modern herders who practice a mixed strategy, possibly implying a greater emphasis on meat production. The increased mortality of camelids between two and three years of age could reflect the selection of young animals or the culling of surplus [66]. On the other hand, fusion groups A and C amount to a few specimens and their confidence intervals are too wide, making it difficult to discriminate between RU1 and other archaeological or actualistic profiles.

Filleting marks, green breaks and the recovery of cooking wares are indicative of *in situ* preparation and consumption. Correlation analysis of long-bone FUI suggests an unbiased selection of skeletal elements, while the strong and negative correlation with the drying index suggest two scenarios: 1. provision of fresh-cuts from carcasses used to produce *chalona*, and, 2. the local production of *chalona* to be consumed elsewhere. This is counterintuitive, as hunting of wild camelids in the Puna would display a positive correlation, and the same as distribution from state deposits. Therefore, local production of *chalona* seems at first glance to be the more parsimonious hypothesis. Dried meat can be produced at a wide range of altitudes below the Highlands or Puna [93]. *Chalona* could have been produced as part of the corvée, since dried meat made from wild camelids was listed as one of the animal products given in tribute to the Inka [10].

## Conclusions

The faunal assemblage from RU1 shows remarkable continuities with sites and localities prior to the Inka Period. Taxonomic diversity on the family and species levels was low and broadly similar to other residential units dated to ca. AD 1200–1536, as well as to most agropastoral sites from semiarid intermontane valleys in northwestern Argentina. The skeletal profile of Camelidae was neither indicative of differential access nor of centralized distribution; negative correlation with the Drying Index could be interpreted in terms of local production of dried meat (*chalona*) for consumption elsewhere. Fusion-based survivorship data showed a mixed pattern and a slight increase of mortality for camelids around three years-old.

In sum, evidence reviewed here does not support the hypothesis of change to state-sponsored distribution for the Inka Period. On the contrary, continuity with the Regional Developments II Period is the most ostensible pattern. Further research on Til1 and other contemporaneous sites is required for evaluation of the results and conclusions reached here.

## Supporting Information

S1 File Identification. Anatomic, taxonomic and taphonomic identification of bone specimens from RU1.
(XLSX)

S2 File Morphometry. Morphometric measurements of camelid specimens from RU1.
(XLSX)
